# Ileocolic intussusception: a case report and literature review

**DOI:** 10.1093/jscr/rjaf301

**Published:** 2025-07-26

**Authors:** Gloria Goi, Claudio Guerci, Luca Ferrario, Giulia Maria Beatrice Lamperti, Francesco Cammarata, Andrea Kazemi Nava, Piergiorgio Danelli

**Affiliations:** General Surgery Department, Luigi Sacco University Hospital, Via G. B Grassi 74, Milan 20157, Italy; University of Milan, Via Festa del Perdono 7, Milan 20122, Italy; General Surgery Department, Luigi Sacco University Hospital, Via G. B Grassi 74, Milan 20157, Italy; General Surgery Department, Luigi Sacco University Hospital, Via G. B Grassi 74, Milan 20157, Italy; General Surgery Department, Luigi Sacco University Hospital, Via G. B Grassi 74, Milan 20157, Italy; University of Milan, Via Festa del Perdono 7, Milan 20122, Italy; General Surgery Department, Luigi Sacco University Hospital, Via G. B Grassi 74, Milan 20157, Italy; University of Milan, Via Festa del Perdono 7, Milan 20122, Italy; General Surgery Department, Luigi Sacco University Hospital, Via G. B Grassi 74, Milan 20157, Italy; General Surgery Department, Luigi Sacco University Hospital, Via G. B Grassi 74, Milan 20157, Italy; University of Milan, Via Festa del Perdono 7, Milan 20122, Italy

**Keywords:** intussusception, colorectal surgery, laparoscopy

## Abstract

Intussusception is a rare cause of intestinal obstruction. In adults, it is often secondary to an underlying pathology. Imaging plays a central role in the diagnosis. Surgical intervention is the treatment of choice when bowel obstruction occurs. The goal of surgery is both therapeutic and diagnostic, allowing for resection and pathological evaluation. This case of a 76-year-old female shows that early recognition and prompt surgery are crucial for a favorable outcome. The patient exhibited symptoms of bowel obstruction. The computed tomography scan clearly demonstrated intussusception of the terminal ileum into the cecum. The patient underwent urgent laparoscopic right hemicolectomy with extracorporeal anastomosis. Histological findings: intussusception with a tubulovillous adenoma with low-grade dysplasia and without invasive features. In this case, an underlying potentially malignant evolving condition was discovered and removed. In conclusion, early recognition and intervention are key to improving outcomes in patients with intussusception and intestinal obstruction signs.

## Introduction

Intussusception in adults is a rare cause of intestinal obstruction, accounting for only 1%–5% of all cases of obstructions and less than 5% of all intussusceptions [1]. Unlike pediatric cases, where intussusception is commonly idiopathic, in adults, it is often secondary to an underlying pathology, even malignancy, which is identified in up to 65% of cases [2]. Other causes include benign lesions, like lipomas, adenomas, Meckel’s diverticulum, and postsurgical adhesions. Inflammatory conditions, such as Crohn’s disease, can also predispose individuals to intussusception [3].

Adult intussusception often presents with nonspecific symptoms, including abdominal pain, nausea, vomiting, and signs of bowel obstruction.

Imaging plays a central role in the diagnosis of intussusception. Abdominal computed tomography (CT) with contrast is considered the gold standard due to its high sensitivity and specificity. In adult cases, CT typically shows the characteristic “target” or “sausage-shaped” mass, which reflects the telescoping of one bowel segment into another [4].

Surgical intervention is the treatment of choice for adult intussusception, particularly when there is evidence of bowel obstruction or suspicion of malignancy. The goal of surgery is both therapeutic and diagnostic, allowing for resection of the affected bowel and pathological evaluation [5].

This case of a 76-year-old female presenting with an occlusive syndrome due to ileocolic intussusception illustrates the complexity and rarity of the condition in the adult population. Early recognition and prompt surgical intervention are critical for a favorable outcome, especially when there is suspicion of underlying pathology, which often occurs in adult patients.

Laparoscopic surgery offers the advantage of a minimally invasive approach, reducing postoperative pain, hospital stay, and recovery time compared to open surgery [6].

## Case report

**Figure 1:**
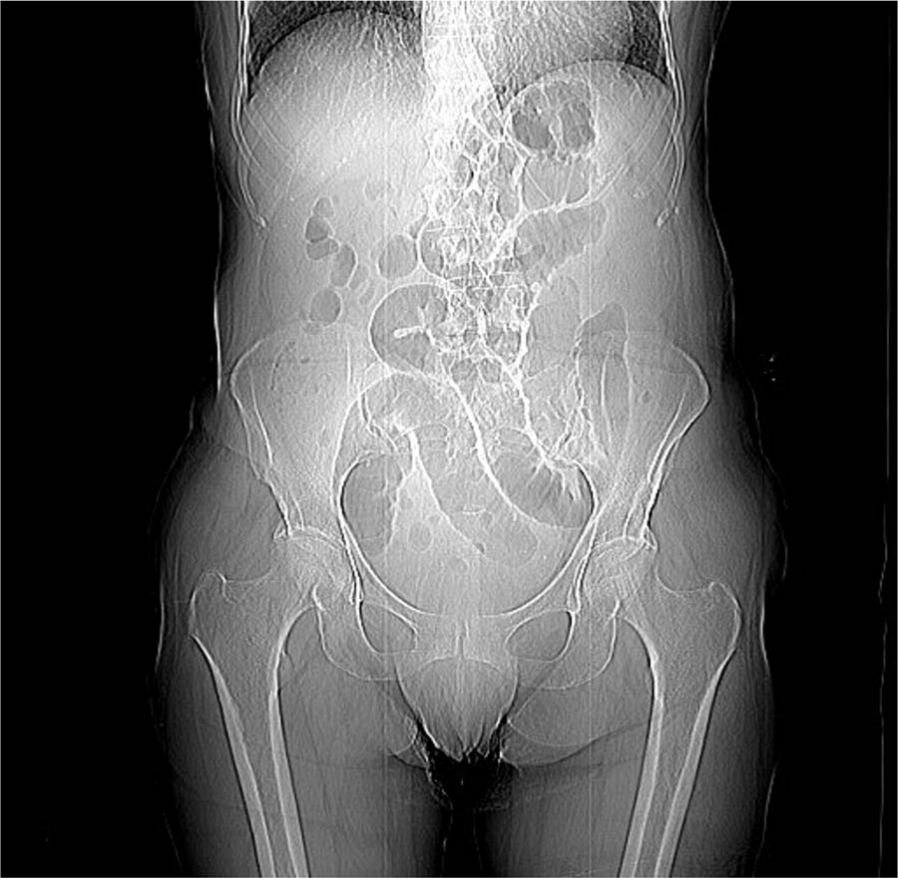
Coronal abdominal RX.

**Figure 2:**
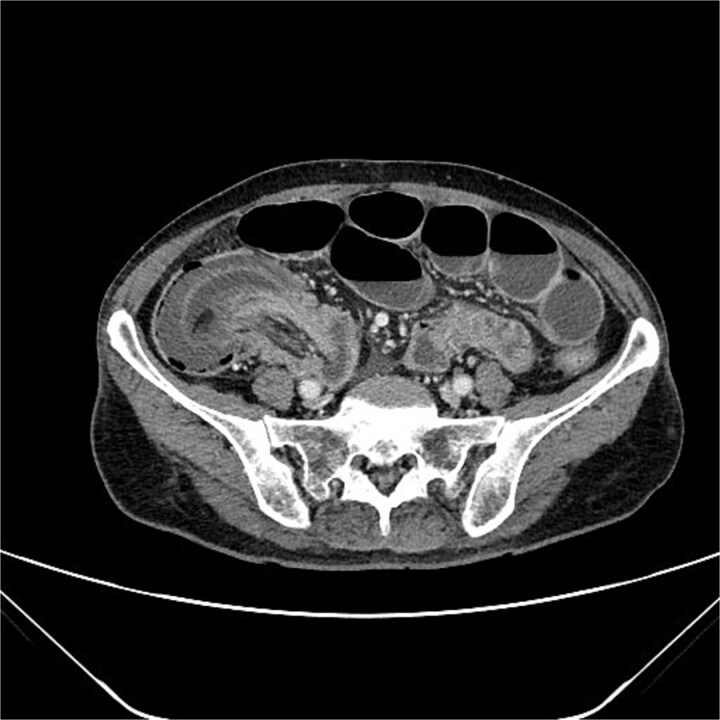
Axial abdominal CT scan.

**Figure 3:**
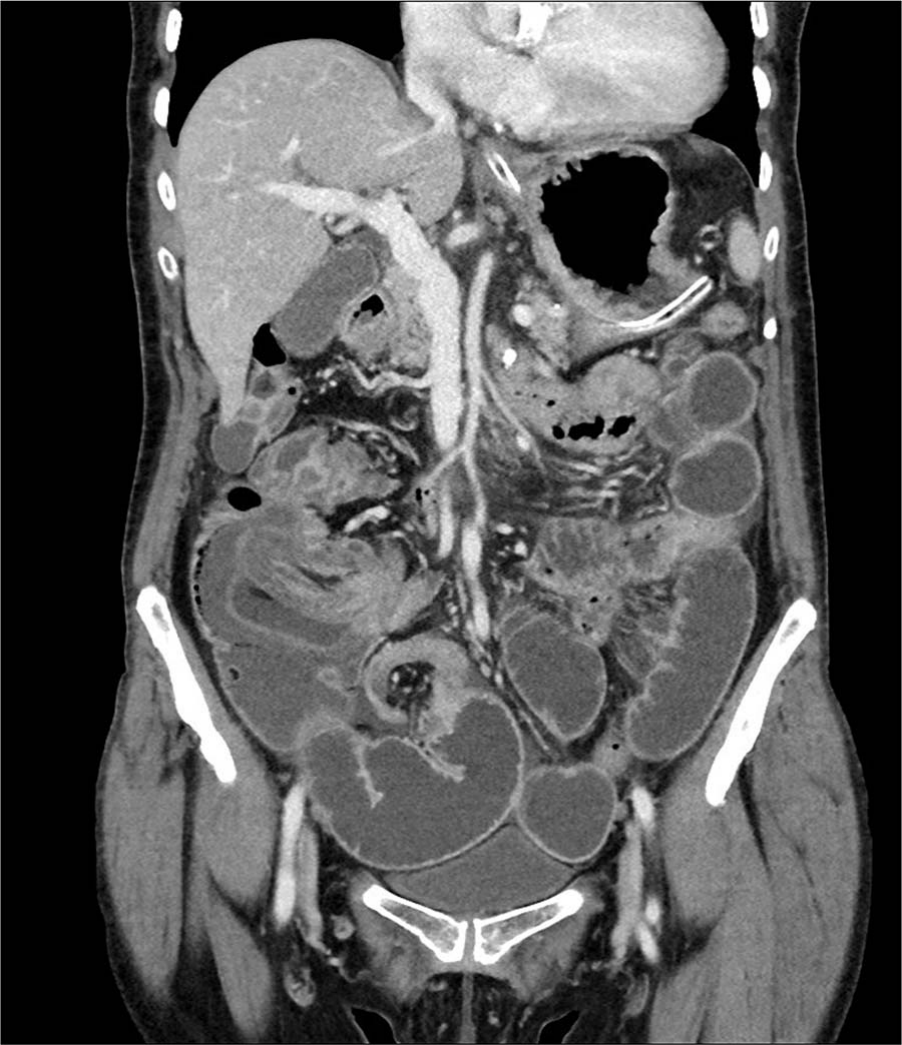
Coronal abdominal CT scan.

A 76-year-old female patient presented to the emergency department with abdominal pain and vomiting. A standard radiography (Fig. 1) and a contrast-enhanced abdominal CT scan revealed an intestinal obstruction caused by intussusception of the terminal ileum into the cecum (Figs 2 and 3). Laboratory tests showed elevated C-reactive protein levels and leukocytosis. Her medical history was significant for bilateral carotid artery stenosis (under treatment with clopidogrel), mild obstructive sleep apnea syndrome, and hypertension.

The patient exhibited classical symptoms of bowel obstruction (abdominal pain and vomiting), confirmed by imaging studies. The elevation of inflammatory markers suggested a more advanced disease stage, possibly with localized inflammation or ischemia. The patient’s prior medical history did not directly influence the intussusception, but these comorbidities required careful perioperative management. The CT scan clearly demonstrated intussusception of the terminal ileum into the cecum, which is consistent with an ileocolic intussusception, a relatively common form in adults. Given the clinical and radiological findings, the patient underwent urgent laparoscopic right hemicolectomy with extracorporeal anastomosis.

The 47-cm-long specimen was examined. On opening the specimen, a 4-cm-large and solid neoplasia was identified, limited to the superficial layers of the cecum wall. The histopathological study revealed a tubulovillous adenoma of the large intestine with low-grade dysplasia and without invasive features, a segment of small intestine with prolapsed, ulcerated, and necrotic mucosa, expression of a subacute ischemic ileitis due to intestinal intussusception and obstruction.

This case report has been described in accordance with SCARE criteria [7].

## Discussion

Intussusception is described as the telescoping of one bowel segment with its mesenteric fold into an adjoining bowel tract, causing venous congestion and blood supply reduction. Intussusception can occur anywhere along the small and large bowel. This condition in adults poses diagnostic and therapeutic challenges due to its rarity and the nonspecificity of symptoms. Most adult cases are associated with an underlying lesion, often neoplastic, benign [8] or malignant (metastatic lesions, lymphomas, and adenocarcinomas), which underscores the importance of surgical resection and histological analysis [5]. The use of contrast-enhanced CT has greatly improved the preoperative diagnosis of intussusception, allowing for early and accurate identification of the condition. CT scan shows a peculiar sign, described either as “target,” “bulls- eye,” or “sausage-shaped” lesion (Fig. 3). This pathognomonic sign can be identified at coronal and axial view [5]. This case emphasizes the need for a high index of suspicion in elderly patients presenting with bowel obstruction and nonspecific abdominal symptoms. Prompt surgical intervention, as performed in this case, is crucial to prevent complications such as bowel ischemia or perforation, which significantly increase morbidity and mortality. Moreover, in this case, an underlying potentially malignant evolving condition was discovered and removed. Given the patient’s overall clinical status and the urgency of the situation, the choice of surgical management was appropriate.

In conclusion, adult intussusception is a rare but serious condition that requires timely diagnosis and surgical management. The use of modern imaging techniques, such as CT, is essential for preoperative planning, while surgery remains the definitive treatment, often revealing the underlying cause. In this case, laparoscopic right hemicolectomy was successfully performed, and histopathological results will further clarify the etiology. Early recognition and intervention are key to improving outcomes in these patients.
